# Novel strategies of Raman imaging for brain tumor research

**DOI:** 10.18632/oncotarget.19668

**Published:** 2017-07-28

**Authors:** Imiela Anna, Polis Bartosz, Polis Lech, Abramczyk Halina

**Affiliations:** ^1^ Lodz University of Technology, Institute of Applied Radiation Chemistry, Laboratory of Laser Molecular Spectroscopy, 93-590 Lodz, Poland; ^2^ Polish Mother’s Memorial Hospital Research Institute, Department of Neurosurgery and Neurotraumatology, 3-338 Lodz, Poland

**Keywords:** Raman spectroscopy, Raman imaging, brain tumor, CNS, iodine number

## Abstract

Raman diagnostics and imaging have been shown to be an effective tool for the analysis and discrimination of human brain tumors from normal structures. Raman spectroscopic methods have potential to be applied in clinical practice as they allow for identification of tumor margins during surgery. In this study, we investigate medulloblastoma (grade IV WHO) (n= 5), low-grade astrocytoma (grades I-II WHO) (n =4), ependymoma (n=3) and metastatic brain tumors (n= 1) and the tissue from the negative margins used as normal controls. We compare a high grade medulloblastoma, low grade astrocytoma and non-tumor samples from human central nervous system (CNS) tissue. Based on the properties of the Raman vibrational features and Raman images we provide a real–time feedback method that is label-free to monitor tumor metabolism that reveals reprogramming of biosynthesis of lipids, proteins, DNA and RNA. Our results indicate marked metabolic differences between low and high grade brain tumors. We discuss molecular mechanisms causing these metabolic changes, particularly lipid alterations in malignant medulloblastoma and low grade gliomas that may shed light on the mechanisms driving tumor recurrence thereby revealing new approaches for the treatment of malignant glioma. We have found that the high-grade tumors of central nervous system (medulloblastoma) exhibit enhanced level of β-sheet conformation and down-regulated level of α-helix conformation when comparing against normal tissue. We have found that almost all tumors studied in the paper have increased Raman signals of nucleic acids. This increase can be interpreted as increased DNA/RNA turnover in brain tumors. We have shown that the ratio of Raman intensities I_2930_/I_2845_ at 2930 and 2845 cm^-1^ is a good source of information on the ratio of lipid and protein contents. We have found that the ratio reflects the different lipid and protein contents of cancerous brain tissue compared to the non-tumor tissue. We found that levels of the saturated fatty acids were significantly reduced in the high grade medulloblastoma samples compared with non-tumor brain samples and low grade astrocytoma. Differences were also noted in the n-6/n-3 polyunsaturated fatty acids (PUFA) content between medulloblastoma and non-tumor brain samples. The content of the oleic acid (OA) was significantly smaller in almost all brain high grade brain tumors than that observed in the control samples. It indicates that the fatty acid composition of human brain tumors differs from that found in non-tumor brain tissue. The iodine number N_I_ for the normal brain tissue is 60. For comparison OA has 87, docosahexaenoic acid (DHA) 464, α-linolenic acid (ALA) 274. The high grade tumors have the iodine numbers between that for palmitic acid, stearic acid, arachidic acid (N_I_=0) and oleic acid (N_I_=87). Most low grade tumors have N_I_ similar to that of OA. The iodine number for arachidonic acid (AA) (N_I_=334) is much higher than those observed for all studied samples.

## INTRODUCTION

Tumors of the central nervous system (CNS) are the most common tumors in children and adolescents (0-19 years old). In adults (20+) the most frequent tumor are non-Hodgkin lymphoma and melanoma, with CNS tumors on the third place. It was estimated that in 2014 in US alone 4,600 individuals (0-19) will be diagnosed with cases of primary CNS tumor [1]. World Health Organization (WHO) described more than 120 distinct types of CSN tumors [2]. The principal goal is complete removal of the tumor; therefore, it is essential to know the tumor borders. In most high-malignant gliomas, the tumor boundaries due to infiltration are impossible to define exactly. According to WHO recommendations and updated in 2016 with the molecular genetic testing [3], main groups of tumors of the CNS classified on the basis of the cells’ origin and location in the brain are: diffuse astrocytic and oligodendroglial tumors, ependymal tumors, choroid plexus tumorous, neuronal and mixed neuronal-glial tumors, tumors of the pineal region, embryonal, tumors of the cranial and paraspinal nerves, meningiomas, mesenchymal non-meningothelial tumors, melanocytic tumors, lymphomas, histocytic tumors, germ cell tumors, tumor of the sellar region, and metastatic tumors. All tumors are characterized according to abnormal tumor tissue appearance (evaluated under a microscope and with immunohistochemical and genetics analysis), and categorized on the basis of the tumor grade classification (I-IV grades, depending on the tissue abnormality) [4]. Approximately half of primary CNS tumors are gliomas, which are further categorized as low-grade astrocytoma (grade I and II), anaplastic astrocytoma (grade III) and glioblastoma multiforme (grade IV). This grading is subjective so particular tumors often do not represent any above listed grade [5]. The most common are tumors of the pituitary and astrocytomas [6]. There are several types of treatment such as chemotherapy, immunotherapy, radiation or targeted therapy, but the basic one is a surgery [7]. Knowledge about the type of tumor and its location is crucial to further treatment and patient survival. Identification of the type of cancer is commonly carried out by cytology, histopathology, immunohistochemistry and genetics. Over the years. various diagnostic imaging techniques have been developed for CNS imaging and to delineate the excision border such as radiography, ultrasonography (USG), computed tomography (CT), magnetic resonance imaging (MRI), neuronavigation and neuromonitoring are routinely used for tumor imaging [8]. However, methods currently used in clinical diagnostics and imaging are very expensive, time consuming and often imperfect because of the limited sensitivity, specificity, spatial resolution and limited intraoperative availability [9]. Most conventional methods of imaging have the spatial resolution that is insufficient to precision required by modern surgical procedures [9]. The MRI imaging is primary method for detection of brain lesions but it has limited capability of distinguishing tumor grade [10]. Moreover, the observed since the late 90's slowdown and side effects of chemotherapy and radiotherapy clearly indicate the need to develop new techniques that may be used in diagnostics, therapy and precise determination of the safety margin removed during surgery. Highly advisable is therefore seeking new methods breaking the previous limitations and with potential clinical applications in order to significantly improve patient intraoperative survival and follow up.

Reports of world literature in recent years clearly indicate that the particular role in the development of innovative techniques for medical diagnostics and molecular imaging is played by methods of vibrational spectroscopy (IR and Raman), among which the dominant role belongs to Raman imaging, which allow simultaneous monitoring of morphological and biochemical properties with very high spectral and spatial resolution [11–14].

The vibrational imaging (Raman, IR) is capable of simultaneously recording of mapping multiple regions and providing spatial distribution of proteins, lipids, nucleic acids, and metabolites within the CNS. Raman imaging and spectroscopy may cause a revolution in the diagnosis and treatment of tumor by providing information about the spatial location of biochemical components in cell organelles, in contrast to the classical methods LC/MS, NMR, HPLC, based on the analysis of samples in the mass subjected to homogenization that prevents spatial characteristics of the systems investigated.

Several groups have investigated the use of Raman spectroscopy to discriminate human and animal brain tissue [9, 15–21] in human xenograft in mice [18], pig brain [15], brain tissue during surgery [19], brain both *in vitro* and in living mice [16]. In particular, Alfano *et al* [11] and Gajjar *et al.* [16] reported the ability of Raman spectroscopy to differentiate between brain tumor and healthy brain tissue.

However, they used human tissue sections obtained from commercially available formalin fixed paraffin preserved (FFPP) tissue blocks. We have shown [22] that the lipid phenotype of tumor in FFPP samples is disturbed because tissue processing (de-waxing, washing and clearing with solvents (e.g. xylene)) has essential effect on loss of cellular lipid constituents. To obtain reliable results the tissue processing method cannot interfere with vibrational spectra of the analyzed tissue. In this study, we use fresh tissue to investigate a brain tumors: a high grade (IV WHO) medulloblastoma, low grade astrocytoma (I and II WHO) and non-tumor samples from human brain tissue. We study human brain tissues with Raman spectroscopy and Raman imaging that will allow diagnostic discrimination of tumors. Based on the properties of the Raman vibrational features, and Raman images we provide a real–time feedback method that is label-free to monitor tumor metabolism. The brain metabolism reveals significant reprogramming of biosynthesis of lipids, proteins, DNA and RNA in diseased states. Our results indicate different metabolic pathways between low and high grade brain tumors. Understanding the biology of metabolic differences in brain tumors may significantly improve patient survival. We discuss molecular mechanisms causing these metabolic changes, particularly lipid alterations in malignant medulloblastoma and low grade gliomas, that may provide insight of the mechanisms driving tumor recurrence. Such knowledge may lead to new approaches for the treatment of malignant glioma.

## RESULTS AND DISCUSSION

**Figure 1:**
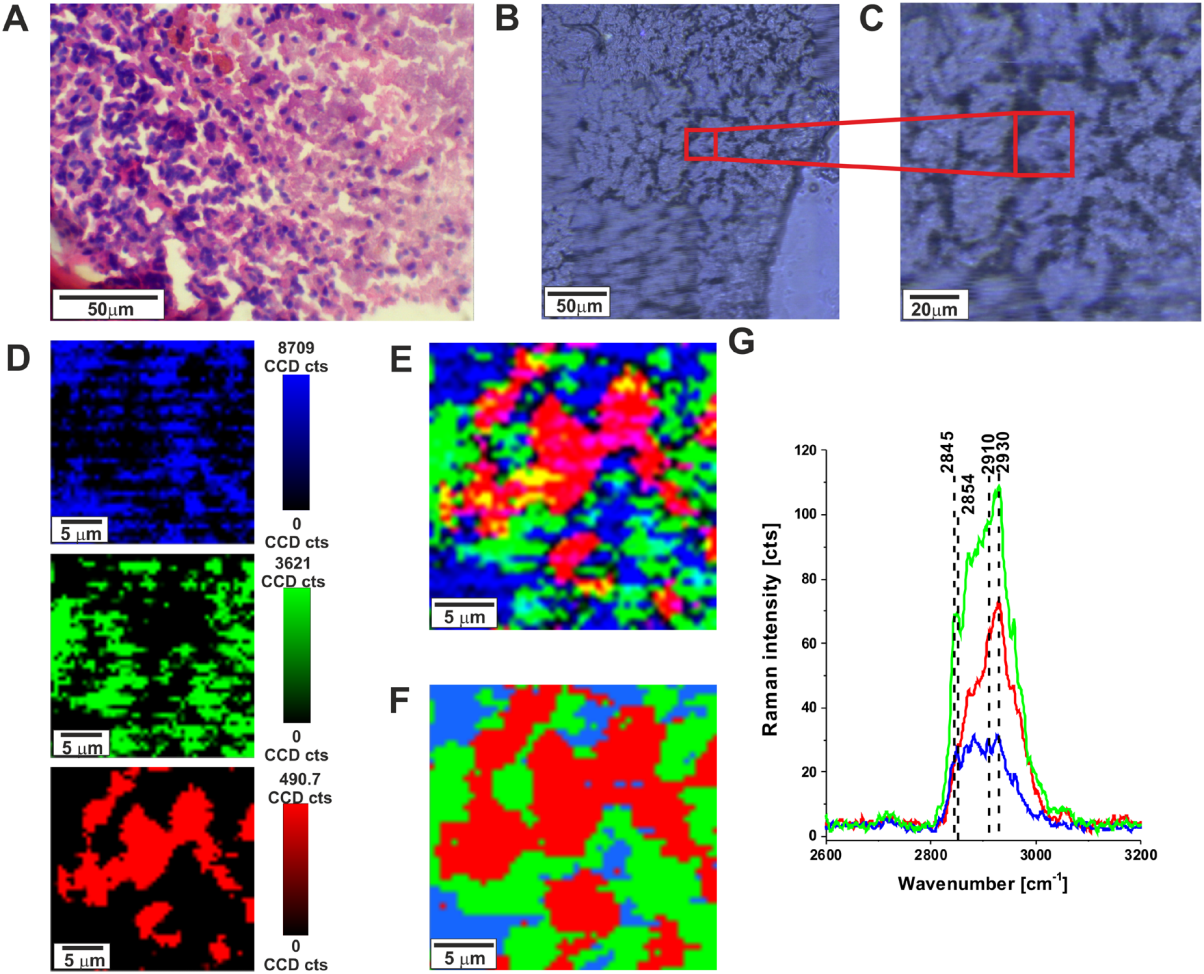
H&E-stained histological image **(A),** stitching microscopy image (250 μm x 270 μm) **(B),** microscopy image **(C)** distribution of compounds in three spectral ranges 2840-2960 cm^-1^ (blue),2900-2910 cm^-1^ (green), 2920-2950 cm^-1^ (red) **(D)** Raman images (25 μm x 25 μm) obtained from basis analysis **(E)** and cluster analysis **(F)** as well as the characteristic vibrational Raman spectra **(G)** in the high frequency region of the normal brain tissue from the negative safety margin (right) (P17), integration time for Raman images 0.5 s, resolution step: 0.5 μm, laser excitation power: 10 mW. The line colors of the spectra correspond to the colors of the Raman maps.

To quantitatively evaluate Raman imaging as a diagnostic method for identifying tumor infiltration in specific regions of brain, we compared tumor detection via Raman imaging, white light microscopy imaging and H&E stained microscopic imaging. Figure 1 shows the H&E-stained and non-stained microscopic image, Raman images, and vibrational Raman spectra in the high frequency region of the normal brain tissue (P17). One can see that both Raman microscopy, conventional microscopy and H&E staining are capable of generating highly correlated images of the microscopic architecture of tissues.

After verifying that Raman microscopy accurately captures the microscopic architecture of normal brain structures it is important to emphasize that, unlike H&E, where contrast is generated by the binding of H&E to a range of intracellular and extracellular molecules, contrast in Raman microscopic images provide information about biochemistry of the tissue itself. The Raman signal intensities in the fingerprint region and high frequency region at around 2845- 2960 cm^−1^are related to various chemical components of the tissue. The molecular basis of contrast in Raman microscopy explains why it is well suited for differentiating cellular brain regions. Differences in Raman spectra among specific brain regions reflect different compositions of lipid-rich and protein-rich regions. Figure 1G shows the Raman spectra in lipid-rich regions (blue color), protein-rich region (red color) and mixed lipid-protein profile (green color) in human normal brain. We have shown in our previous papers [23, 24] that the Raman peaks at 2845 and 2854 cm^-1^ are characteristic vibrational features for lipids whereas the peak at around 2930 - 2940 cm^-1^ is characteristic for proteins. Brain white matter is lipid-rich, tumors are protein-rich and a mixed profile is characteristic for cortex with acellular, myelin-rich regions such as fiber tracts [17]. Therefore, the ratio of Raman intensities I_2930_/I_2845_ at 2930 and 2845 cm^-1^ is accepted as a good source of information on the ratio of lipid and protein contents. Table 1 shows the Raman intensity ratios I_2930_/I_2845_ for all analyzed brain tissue samples.

**Table 1:** Raman intensity ratios at 2930/2845 cm^-1^ for all analyzed brain tissue samples

Type of cancer	Patient number	Intensity at 2930 cm^-1^	Intensity at 2845 cm^-1^	Ratio I_2930_/I_2845_	Standard deviation
Medulloblastoma WHO grade IV	1	0.07	0.09	0.78	0.010
	2	0.09	0.04	2.32	0.026
	3	0.12	0.05	2.55	0.035
	4	0.10	0.06	1.68	0.021
	9	0.10	0.04	2.62	0.032
Astrocytoma WHO grade II	7	0.11	0.04	3.15	0.038
	8	0.10	0.06	1.77	0.022
Astrocytoma WHO grade I	10	0.11	0.04	2.977	0.037
	14	0.11	0.04	2.63	0.034
Ependymoma WHO grade II	5	0.09	0.04	2.06	0.028
	6	0.08	0.05	1.74	0.018
	11	0.12	0.03	3.81	0.043
Ganglioma WHO grade II	13	0.11	0.06	1.83	0.024
Heamaningioblastoma WHO grade I	15	0.11	0.06	0.87	0.025
Metastatic brain tumor	16	0.11	0.05	1.96	0.026
Normal brain tissue	17	0.108	0.07	1.46	0.016

The ratio reflects the different lipid and protein contents of cancerous brain tissue compared to the non-tumor tissue. One can see that almost all studied brain tumors have the ratios significantly higher than found in normal brain tissue, which is 1.456 ± 0.016. It indicates that the relative amount of lipids compared to proteins is markedly higher in the normal brain tissue. Our results are in agreement with the results presented in [25] where the fatty acid composition of tumor and tumor-free brain tissue from glioma patient was measured using chromatography techniques. They found that levels of the polyunsaturated fatty acid (PUFA) docosahexaenoic acid (DHA) were markedly reduced in tumor samples in comparison with non-tumor brain. This reduction of DHA content was also observed in connection with reduction of phospholipids (phosphatidylserine and phosphatidylethanolamine) content for the glioma samples. Differences were observed in the n-6 PUFA content between tumor and non-tumor brain samples. The n-6 PUFA linoleic acid content in terms of total lipids composition was significantly greater in tumor tissue than in normal one. Thus, the fatty acid composition of human gliomas differs from that found in non-tumor brain tissue [25].

**Figure 2:**
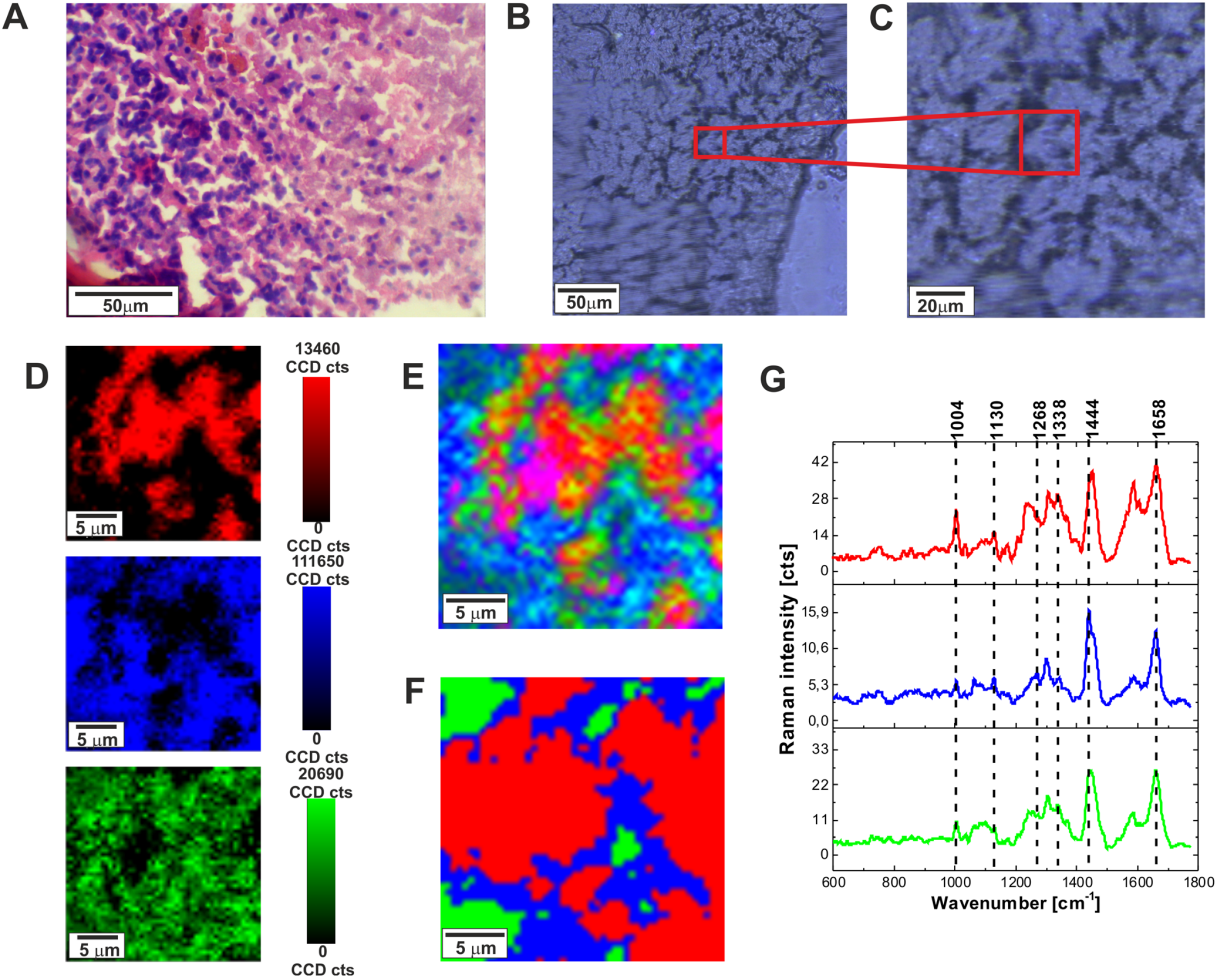
H&E-stained histological image **(A)**, stitching microscopy image (250 μm x 270 μm) **(B)**, microscopy image **(C)**, distribution of compounds in three spectral ranges **(D)** Raman images (25 μm x 25 μm) obtained from basis analysis **(E)** and cluster analysis **(F)**, as well as the characteristic vibrational Raman spectra **(G)** in the low frequency region of the normal brain tissue from the negative safety margin (right) (P17), integration time for Raman images 1 s, resolution step: 0.5 μm, laser excitation power: 10 mW. The line colors of the spectra correspond to the colors of the Raman maps.

Raman fingerprint region can provide additional valuable information about the biochemistry of brain. Figure 2 shows the H&E-stained and non-stained microscopic image, Raman images, and vibrational Raman spectra in the fingerprint region for normal tissue.

**Figure 3:**
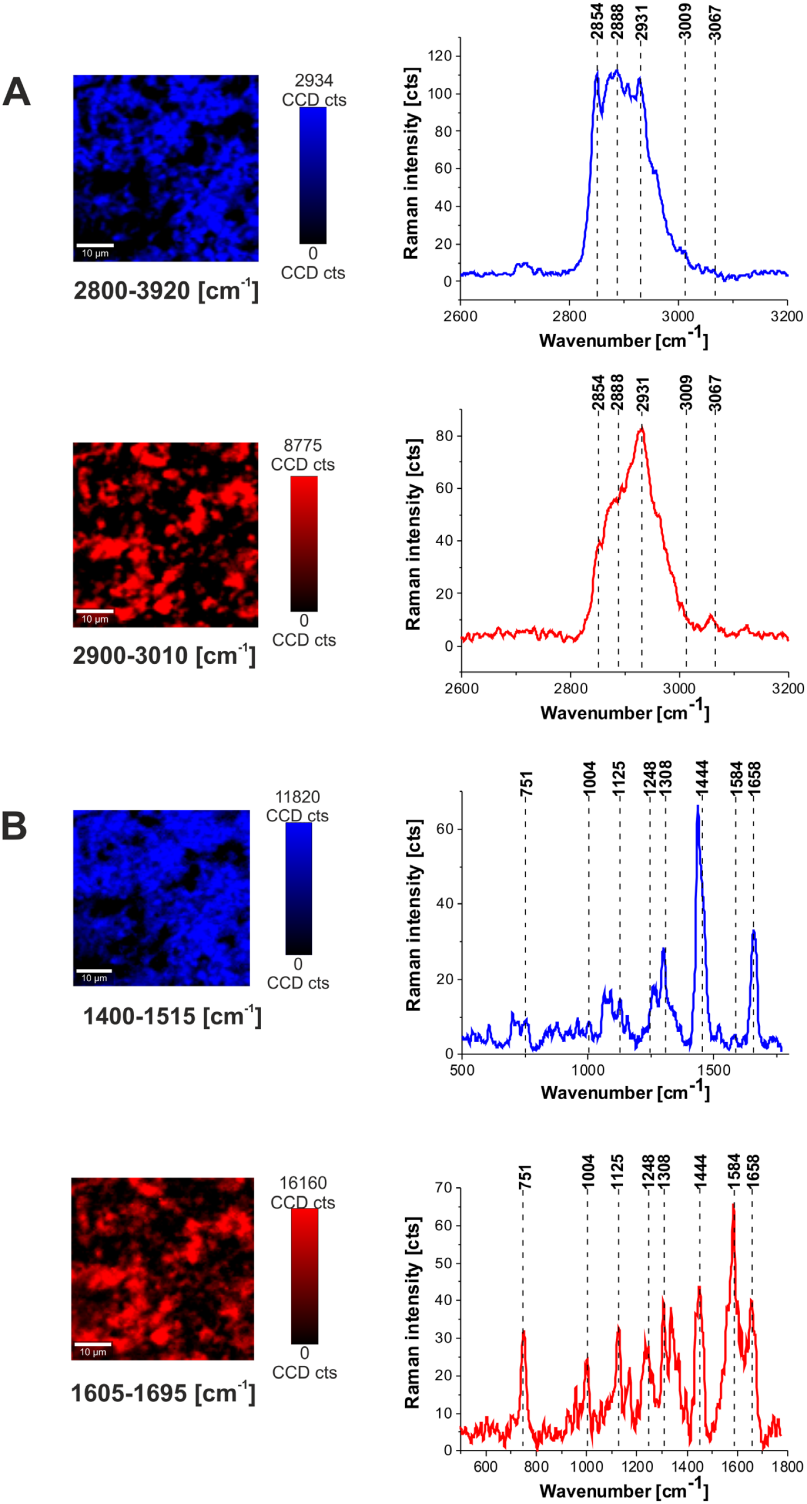
Distribution of lipids and proteins in the human normal brain tissue (P 17) obtained from the basis analysis in the high frequency region and fingerprint region with the following spectral filters **(A)** Two filters 2800-2920 cm^-1^ (lipids) and 2900-3010 cm^-1^ (proteins) for the high frequency region, and **(B)** two filters for the fingerprint region 1400-1515 (lipids), 1605-1695 (proteins).

To identify distribution of biochemical components in the brain tissue we have used Raman images of pure components as spectral filters of corresponding Raman vibrations. To obtain distribution of various components the in the brain tissue the basis analysis method was used on Raman images. Figure 3 shows distribution of lipids and proteins in the human normal brain tissue (P17) obtained from the basis analysis in the high frequency region and fingerprint region with the following spectral filters: (a) two filters 2800-2920 cm^-1^ (lipids) and 2900-3010 cm^-1^ (proteins) for the high frequency region, and (b) two filters for the fingerprint region 1400-1515 (lipids), 1605-1695 (proteins).

Figure 3 shows that Raman images created both for high frequency and fingerprint regions show almost identical distribution of lipids and proteins, which additionally proves the correctness of the applied procedure. As one can see from Figure 3 the Raman spectra in the fingerprint region are very sensitive to cellular environment and the distribution of various components is inhomogeneous. Since inhomogeneity is a common feature of most tumors, made up from necrotic cores, proliferative rims and infiltrations into surrounding tissue, the spectrum may vary greatly depending on the Raman imaging sampled region. Hence, the region of chosen for analysis will have a large impact on the results. Therefore, it some cases it is useful to analyze the average Raman spectra.

**Figure 4:**
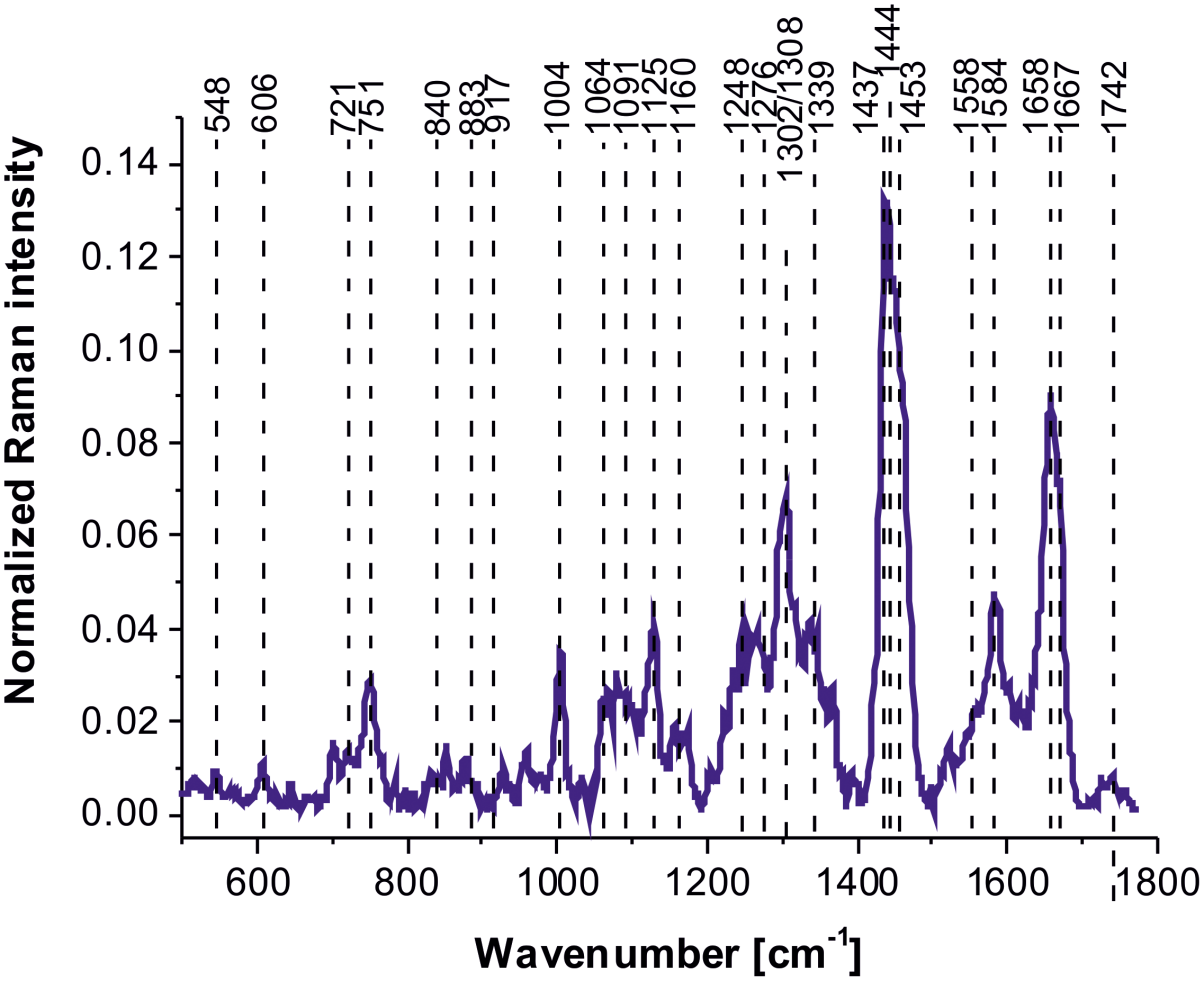
Average Raman spectrum of the human normal brain tissue in the fingerprint region

Figure 4 shows average vibrational Raman bands of the human normal brain tissue in the fingerprint region chosen in a randomized fashion over the sample to avoid any bias.

Table 2 shows tentative assignments of vibrational Raman bands of the normal human brain tissue from the average Raman spectra. Detailed inspection into Figure 4 and Table 2 demonstrates that the Raman spectra of the human normal brain tissue provide information about proteins (α-helix proteins at ∼1658 cm^-1^ (Amide I), ∼1276 cm^-1^ (Amide III); β-sheet proteins at ∼1667-1680 cm^-1^ (Amide I), 1558 cm^-1^ (Amide II), 1238 cm^-1^ (Amide III), 2940 cm^-1^ (CH_3_ stretching)), lipids (cell membrane phospholipids at ∼1080-1158, 1248 cm^-1^ (symmetric and antisymmetric P=O stretching), fatty acids, triglycerides at ∼1437-1444 cm^-1^ (CH_2_ deformation(bending)), at ∼1658 (C=C stretching), at ∼1742 cm^-1^ (C=0 stretching), ∼2845-2940 cm^-1^ (CH_2_, CH_3_ stretching)), (H3C)N^+^) choline group can at ∼721 cm^-1^), nucleic acids (∼751 cm^-1^, 1080-1158 cm^-1^, 1584 cm^-1^), and metabolites (glycogen at 840 cm^-1^, lactic acid at 917 cm^-1^).

**Table 2:** Tentative assignments of the vibrational bands of the human CNS from the Raman spectra (patient P17)

Human normal CNS Wavenumber / cm^-1^	Model systems wavenumber / cm^-1^	Tentative assignments
606		Undefined
721	729	Phospholipid (choline)[26-28]
751		Nucleic acids, Trp
840		Tyr, proline, glycogen [16]
883	880	Tyr, Lipids/Carbohydrates/Collagen [26] C-C-N^+^, C-O-C ring, C-C
917		C-C stretch of proline, glucose, lactic acid [16]
958	935	Hydroxyproline/Collagen backbone [27-28] CH=CH bending
997		C-C symmetric stretching, glucose-I-phosphate, sym. breathing mode of phenylalanine [16]
1004 (R)	1004	Phenylalanine [13, 29, 30] sym. ring breathing of protein [31]
1064	1068	Lipids/Collagen [26, 27] C-C str.
1091	1096	Phospholipids, O-P-O sym. str.[27], P=O sym. from nucleic acids/cell membranę phospholipids
1080-1158	1158	Proteins (C-C/C-N str.)[26, 32, 33], P=O sym. from nucleic acids and phospholipids
	1160	L-Tryptophan [31]
1189 (R)	1199	C-C_6_H_5_ Phe, Trp [26]
1238	1240	Phospholipid, O-P-O antisym. Stretch [8] Amide III β–sheet [16]
1276		Amide III [16] α helix, P=O asymmetric stretch due to nucleic acids
1248	1220-1285	Nucleic acids (Try, Ala)/Proteins (Amide III β sheet or random coil), Lipid, phospholipid =C-H bend [26, 27]
1267		Fatty acids, =C-H bend [26, 27]
1304(R)	1304	Lipids, phospholipids [27]C-H_2_ twist, collagen, protein amide III [16], DNA [16]
1339/1370		Trp, C_a_-H def
1437-1444 1453	1444 1461	Fatty acids, triglycerides, CH_2_ or CH_3_ deformations [33] Proteins [30, 31] C-H wag, CH_2_ or CH_3_ def. Phospholipids, CH_2_ scissoring [34]
1558	1556	Amide II, proteins [27, 33], amide II β–sheet [35]
1584	1586	Amide II [16], aromatic amino acids within proteins,[33] nucleic acids [26, 27]
1658	1655	Unsaturated fatty acids, triglycerides (C=C) str.[33], Amide I α helix
1667-1680		Proteins, Amide I β–sheet, cholesterol esters [16]
1667-1680		Proteins Amide I turn [23] / Unsaturated fatty acids [26, 31], (C=O) str., (C-H) def./(C=C) str.[26, 31], collagen, elastin [27]
1732	1743	(C =O) stretching, triglycerides [33]
2845/2854	2854	Fatty acids, triglycerides, C-H_2_ sym. str.
2888	2888	Lipids [31], C-H_2_ antisym. str.
2931/2940	2935	Proteins/Lipids, CH_3_ sym. str.[27, 31]
3009	3008	Lipids [27, 31] =C-H str.
3067	3060	Nucleic acids/Proteins [31] C-H aromatic

Following Raman analysis of the normal brain tissue, spectral data were analyzed for all human brain tumors and compared to normal structures.

**Figure 5:**
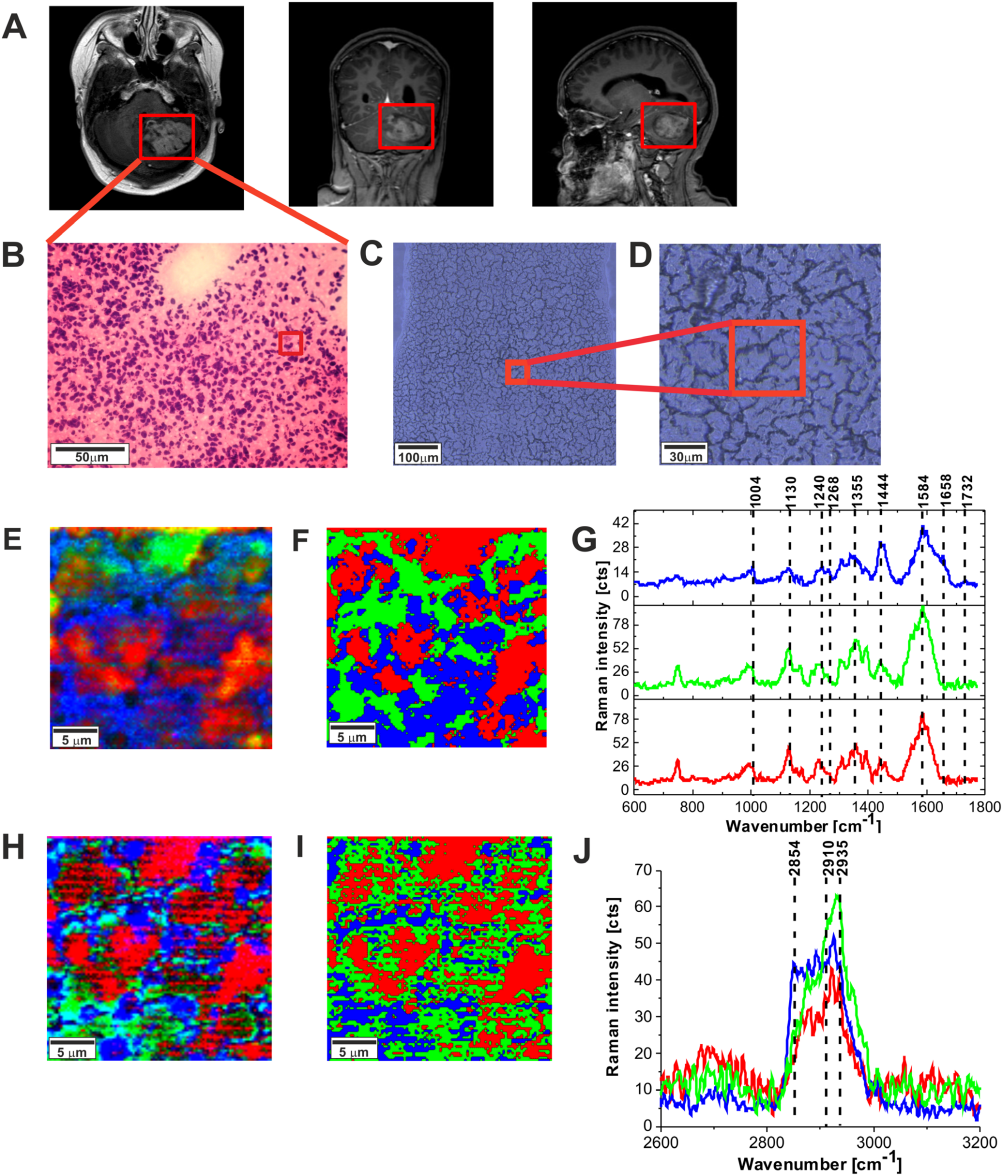
The MRI image **(A),** H&E-stained histological image **(B),** stitching microscopy image (520 μm x 520 μm) **(C),** microscopy image (155μm x 155 μm) **(D)** Raman images (50 μm x 50 μm) obtained by basis analysis **(E)** and cluster analysis **(F)** and the characteristic vibrational Raman spectra in the fingerprint frequency region **(G)** Raman images (50 μm x 50 μm) obtained by basis analysis **(H)** and cluster analysis **(I)** and the characteristic vibrational Raman spectra in the high frequency region **(J)** of the tumor CNS (medulloblastoma, grade WHO IV, infratentorial. Left cerebellar hemisphere) (P9), The line colors of the spectra correspond to the colors of the Raman maps. Raman integration time for images 0.5 s, resolution step: 1 μm, laser excitation power: 10m W.

Figure 5 shows the MRI image, the H&E-stained and non-stained microscopic image, Raman images, and vibrational Raman spectra in the fingerprint region and high frequency regions of the tumor tissue (medulloblastoma, grade WHO IV). One can see that the Raman images of the tumor tissue (Figure 5E and 5F) are dominated by proteins (red region in the Raman images) reflected by Raman peak at 1585 cm^-1^ and 2935 cm^-1^ in Figure 5G in Figure 5J, respectively (detailed analysis of the secondary structure will be discussed below). Lipids (dominated by phospholipids) are located in the membranes (blue colors in Raman images). Comparison between Figure 5E, 5F, 5H, and 5I show that Raman images created both for high frequency and fingerprint regions show almost identical distribution of lipids and proteins, which proves the correctness of the applied procedure.

**Figure 6:**
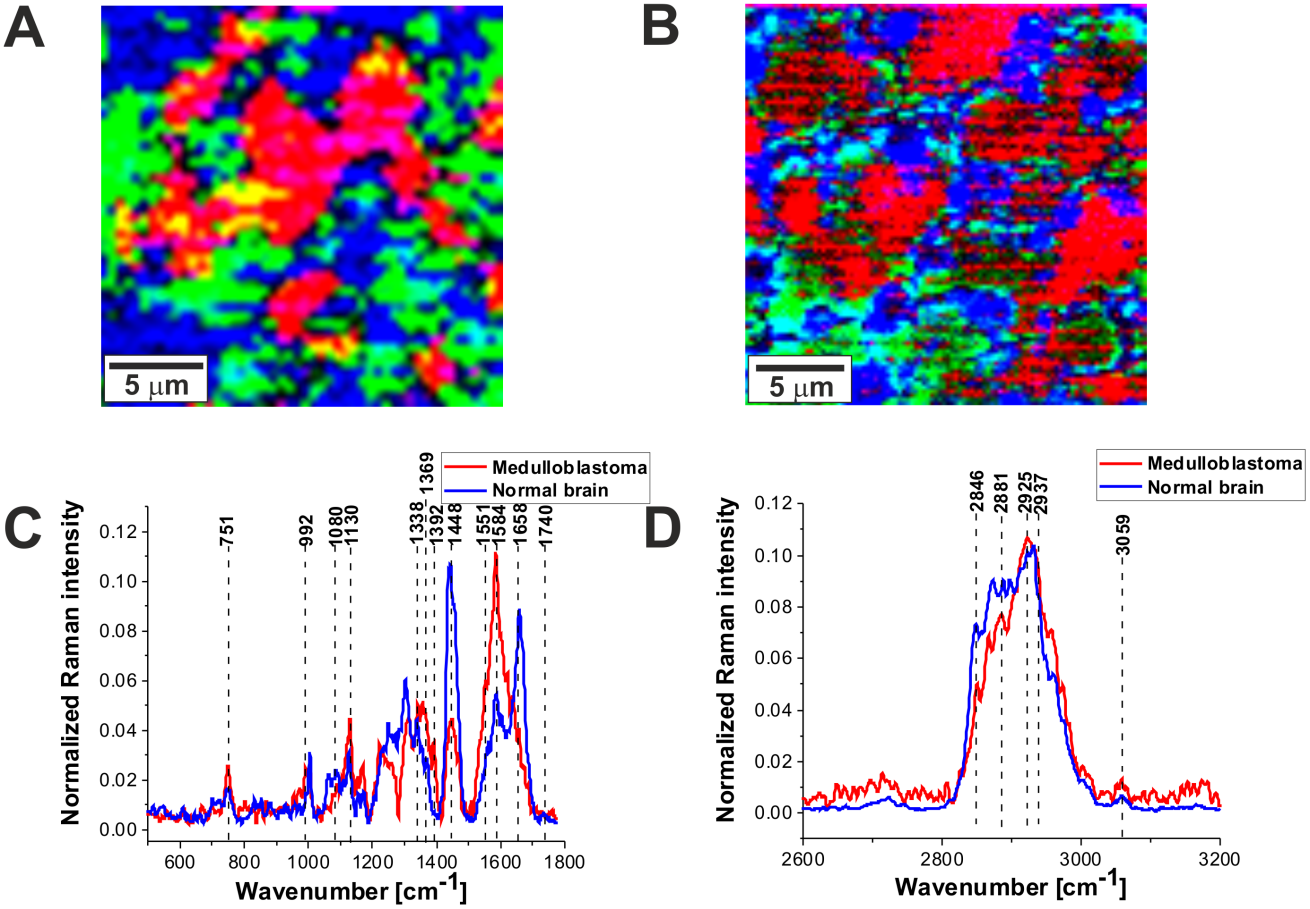
The comparison of the Raman images of the normal **(A)** and tumor (medulloblastoma, grade WHO IV) **(B)** tissues. The average vector normalized Raman spectra in the fingerprint region **(C)** and the high frequency region **(D)** for the high-grade medulloblastoma (medulloblastoma, grade WHO IV, infratentorial. Left cerebellar hemisphere) (P9), compared with the spectra for normal brain.

Figure 6A and 6B shows the comparison of the Raman images of the normal (A) and tumor (medulloblastoma, grade WHO IV) (B) structures. The red and blue areas reflect the location of proteins and lipids, respectively. Figure 6C and 6D shows the average vector normalized Raman spectra in the fingerprint region and the high frequency region for the high-grade medulloblastoma compared with the spectra for normal structure. The Raman spectra for high-grade CNS tumors show many significant differences compared to the Raman spectra of normal structures. Detailed inspection shows notable differences at 751 cm^-1^ (nucleic acids, Trp), 992 cm^-1^ (tyrosine, proline, glycogen, carbohydrates, collagen, glucose, lactic acid), 1080 cm^-1^, 1130/1170 cm^-1^ (phospholipids), 1338cm^-1^ (tryptophan), C_α_-H def), 1369 cm^-1^, 1392 cm^-1^, 1448 cm^-1^ (fatty acids, triglycerides, CH_2_ or CH_3_ deformations), 1551 cm^-1^, 1584 cm^-1^, 1658/1667 cm^-1^ (amide II and amide I, nucleic acids), and 1740- 1759 cm^-1^ (triglycerides)^.^

The most spectacular changes can be observed for Raman bands at 1584 cm^-1^ and 1658/1667 cm^-1^ attributed to proteins/nucleic acids, and amide I, respectively. Our results in Figure 6C shows the intensity of the band of amide I at 1658 cm^-1^ decreases in tumor tissue when comparing against normal tissue. In contrast, the intensity of the band at 1584 cm^-1^ increases indicating that α-helix structure is modified to β-sheet as a result of tumor. This finding is consistent with that of Alfano’s group [36].

A further inspection into Figure 6C shows also an increase in Raman signal intensity between 1230-1250 cm^-1^ in the region of amide III vibrations attributed to β-sheet conformation and an decrease in the region around 1270-1280 cm^-1^ attributed to α-helix conformation. Similar differences in conformational protein structure have been reported earlier [16, 37]. This may suggest that the high-grade tumors of CNS exhibits enhanced level of β-sheet conformation and down-regulated level of α-helix conformation when comparing against normal CNS.

Differences in the second spectral region between tumor and normal tissue are attributed to nucleic acids content. Figure 6C shows that the high grade tumor (medulloblastoma) exhibits markedly higher intensity at 751 cm^-1^ than the normal tissue which indicates that concentration of nucleic acids increases in the high-grade medulloblastoma compared to non-tumor CNS tissue. It was found that nearly all CNS tumors studied in the paper have increased signal of nucleic acids at 751 cm^-1^. This increase can be interpreted as increased DNA/RNA turnover in tumor. The increase at 751 cm^-1^ correlate with a spectacular increase at 1584 cm^-1^ attributed to proteins and nucleic acids.

The third spectral region where notable differences between the normal tissue and the high-grade medulloblastoma tissue are observed is attributed to lipids at around 1437-1444 cm^-1^ (fatty acids, triglycerides, CH_2_ or CH_3_ deformations^11^), andproteins at 1453 cm^-1^ (proteins^10,12^ C-H wag, CH_2_ or CH_3_ def.). Figure 6C shows that the high grade CNS tumor (medulloblastoma) exhibits markedly lower Raman intensity at around 1437-1444 cm^-1^ than the normal tissue which indicates that concentration of saturated CH_2_ bonds in lipids decreases in the high-grade medulloblastoma compared to normal brain tissue. The reduced level of saturated bonds is observed both for the raw Raman spectra and the vector normalized spectra (divided by norm). The same feature of reduced level of lipids is observed for nearly all types of CNS tumors studied in the paper. The results at 1437-1444 cm^-1^ correlate with those presented in Table 1 for the 2845-2854 cm^-1^ attributed to the stretching vibrations of the saturated CH_2_ bonds. However, the normalized intensity of the Raman bands at 721 cm^-1^ (choline), around 1080 cm^-1^ (phospholipid), and 1732 cm^-1^ (triglyceride) increases slightly for almost all types of CNS tumors studied in the paper including the high grade medulloblastoma compared to normal tissue.

Choline bands contains contributions from several different choline-containing compounds, the positive correlation between the 721 cm^-1^ choline signal, and 1080 cm^-1^ phospholipids Raman signals suggests that the increased choline signal in CNS tumors is due to higher levels of phosphocholine (abundantly found in eukaryotic cell membranes as the main membrane compounds such as phosphatidylcholine and sphingomyelin**).** Both choline and phospholipids Raman signals reflect membrane synthesis and degradation in brain tumors due to increased membrane rearrangement. It is well known that great majority of brain tumors have decreased N-acetyl aspartate (NAA) signals, frequently correlated with increased levels of Choline (Cho), leading to increased Cho/NAA ratios [10]. The Cho/NAA ratio is used in human CNS proton magnetic resonance spectroscopy (MRS) in clinical diagnostic [10]. Our Raman results for choline level correlate quite well with the proton magnetic resonance spectroscopy (MRS) results, but also clearly demonstrate that the Raman signals at 1437-1444 cm^-1^ and 2845-2854 cm^-1^ that reflect global level of saturated bonds in lipids (not only choline specific) decreases significantly in high grade brain tumors. The negative correlation of the normalized phospholipid bands (721 cm^-1^, 1080 cm^-1^) and triglycerides at 1732 cm^-1^ with the normalized lipid band at 1437-1444 cm^-1^ and at 2845-2854 cm^-1^ suggests that the decreased level of lipids with saturated bonds (with increased number of C-H_2_ vibrations) is related to significantly different lipid composition due to altered lipid metabolism in brain tumors when comparing against non-tumor structure.

Enhanced anaerobic glycolysis, described by Otto Warburg is one of the most important features of cancer [38]. This process yields an excess of pyruvate, the glycolytic pathway end-product. Majority pyruvate is converted to lactate, with minor part converted to acetyl-CoA, which is used in *de novo* fatty-acid synthesis [39]. Up to now alterations of fatty acid (FA) metabolism in tumor cells have received less attention than glycolysis but there is increasing evidence of importance of altered lipid biosynthesis in tumor metabolism and development [13, 24, 40-63]. It has been recognized recently that the neuronal energy metabolism is not based on glucose only and that fatty acids play an important role in energy metabolism of the central nervous system [44]. Fatty acid uptake and lipid metabolism are deregulated in malignant glioma. Moreover, the recent reports indicate significant metabolic differences between low and high grade gliomas [64]. Although there is no consensus on how fatty acids are transferred into CNS, there is evidence that the albumin-bound fatty acid in plasma is a major contributor to the fatty acid pool in brain, at least in the case of arachidonic acid (AA) and docosahexaenoic acid (DHA) [65].

To address important questions on lipid phenotypic chenges in CNS tumor cells let us remind briefly that metabolic fate of fatty acids can be described as follows: a) oxidation to acetyl CoA for energy production in the form of NADH, b) fatty acid synthase (FASN) used as a key enzyme in the synthetic pathway of fatty acids from acetyl CoA by producing fatty acid palmitate, c) palmityl CoA is a precursor of mono- and polyunsaturated fatty acids, which can be modified into various lipids, such as phospholipids, triglycerides, cholesterol esters, and fatty-acylated proteins, d) Stearoyl-CoA desaturase (SCD) catalyzes further the introduction of double bonds into short-chain FAs at the C9 position (as in converting stearoyl-CoA to oleoyl-CoA) [66], e) fatty acids are used in the synthesis of bioactive molecules such as arachidonic and eicosanoid acids as well as f) cholesterol, steroids and steroid hormones, g) excess fatty acids are typically stored as triglycerides in adipose tissue, h) fatty acids can be metabolized to ketone bodies used as fuel in extrahepatic tissues. FASN is reported to be over-expressed in many human epithelial tumors [51, 52] and is involved in de novo synthesis of fatty acids [53].

**Figure 7:**
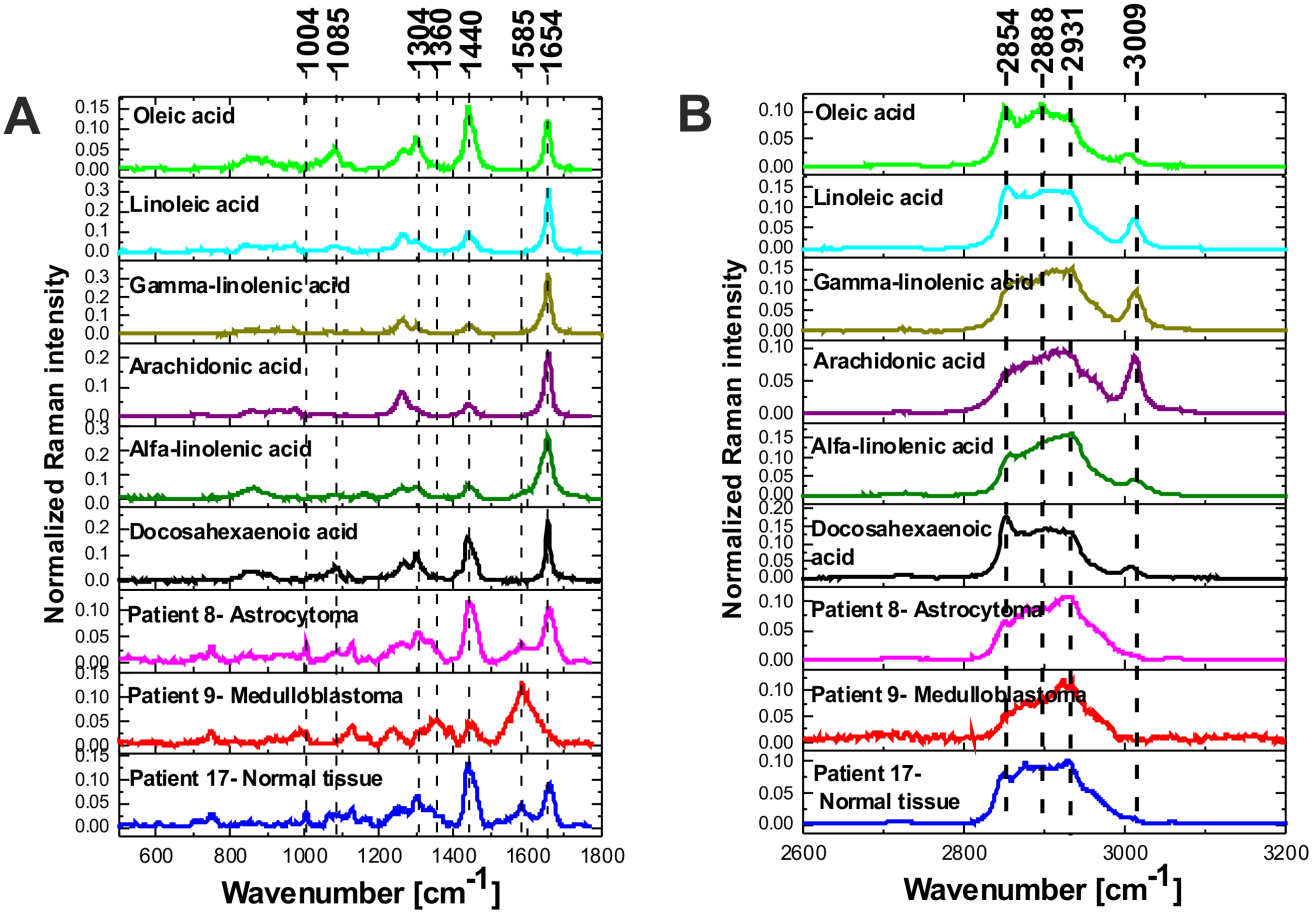
Raman spectra (vector normalized) of PUFA acids compared to the Raman spectra of normal and tumor CNS in fingerprint region **(A)** and high frequency region **(B).**

To understand how lipid constitution changes influence malignant medulloblastoma growth, it is important to first understand the metabolism of fatty acids in normal brain. Lipids make up 2% of the total cell mass in most organs. However, in the CNS, lipids are major structural components with fatty acids constitute about 50% of the total mass of neural membranes [67, 68]. Long chain PUFA such as docosahexaenoic acid (DHA, C22:6, x-3) and arachidonic acid (AA, C20:4, x-6) make up close to 20% of the dry weight of the brain, that include 6% for AA and 8% for DHA [68, 69]. Since the altered metabolism of the PUFA-rich lipid environment of the CNS has been reported to be critical to our understanding of many CNS tumors [69] we will concentrate on the PUFA content in the normal and high grade medulloblastoma tissues by Raman spectroscopy. We will concentrate on the Raman signals at 1437-1444 cm^-1^ and 2845-2854 cm^-1^ that reflect evidently ω reduced global level of saturated bonds of lipids in high grade CNS tumors (Figure 6C and 6D) when comparing against normal tissue. Figure 7 shows the vector normalized Raman intensities of palmitic (17:0), stearic (18:0), oleic OA (18:1, -9), linoleic LA (18:2, ω -6), α-linolenic ALA (18:3, ω -3) that are the precursors of the essential fatty acids from the ω -6 and ω -3 series given also in the plot.

**Figure 8:**
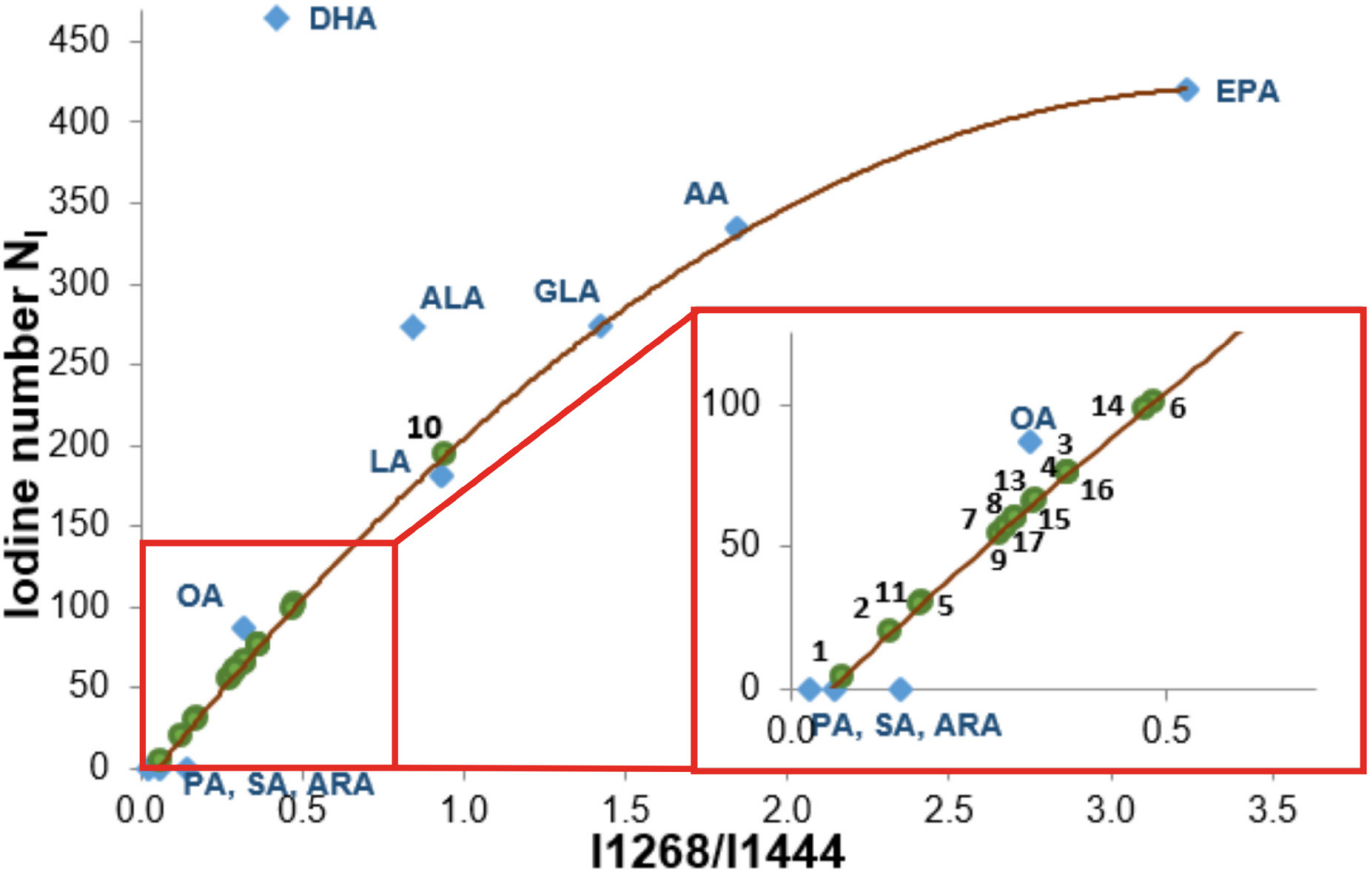
The iodine number N_I_ plot vs. the Raman intensity ratio 1267/1440 for PUFA and brain tissues of studied samples The equation of the curve: y=-37.429x^2^+255.33x-13.618 R^2^=0,9937.

Detailed inspection into Figure 7 shows that the Raman intensity at 1440 cm^-1^ of the normal brain tissue and low grade astrocytoma is nearly identical as for oleic acid OA (and similar to DHA and α-linolenic acid ALA) while the Raman intensity at 1440 cm^-1^ of the high grade medulloblastoma is markedly reduced and nearly identical as for arachidonic acid (AA) and α-linolenic acid. Similar results were obtained for the high frequency range (Figure 7B). The normalized Raman profile for the high grade medulloblastoma is almost perfectly fitted by ALA and AA profiles. However, the marked difference can be seen at 3009 cm^-1^ (C=C-H vibration) because this peak is not present in the CNS tissue. Our results demonstrate that while Raman imaging is undoubtedly highly sensitive modality available for the detection of CNS tumors, several different PUFA types may share a similar Raman spectra appearance, like OA and DHA, or AA and α-linolenic acid. The so called iodine number may be helpful to distinguish between OA and DHA, or AA and ALA. Iodine number increases proportionally to the number of C=C bonds, making it a good source of information on the amount of unsaturation in fatty acids [70]. We have applied the iodine number plot vs. the Raman intensity ratio 1267/1440 used as a good source of information on the ratio of saturated/unsaturated lipids in the human tissue. Figure 8 shows the iodine number plot vs. the Raman intensity ratio 1267/1440 for PUFA and brain tissues of studied samples. Table 3 shows the values of iodine number N_I_ for the brain tissues of studied samples.

**Table 3:** The values of iodine number N_I_ for the brain tissues of studied samples

Types of tumor	Patient number	Iodine number N_I_
Medulloblastoma	1	4
	2	19
	3	76
	4	76
	9	56
Ependymoma	5	30
	6	101
	11	29
Astrocytoma	7	54
	8	60
	10	195
	14	98
Ganglioma	13	65
Haemangioblastoma	15	66
Metastatic brain tumor	16	76
Normal tissue	17	60

The plot shows evidently that the Raman intensity ratio 1267/1440 for normal is determined by properties of OA, not DHA or α-linolenic acid ALA. The iodine number N_I_ for the normal brain tissue is 60. For comparison OA has 87, DHA 464, ALA 274. One can see from the plot in Figure 8 that most high grade tumors have the iodine numbers between that for palmitic acid, stearic acid, arachidic acid (N_I_=0) and oleic acid (N_I_=87). Most low grade tumors have N_I_ similar to that of OA. The iodine number for AA (N_I_=334) is much higher than those observed for all studied samples.

*Cis*-linoleic acid (LA, 18:2, ω -6) and α-linolenic acid (ALA, 18:3, ω -3) are the precursors of AA and DHA, respectively. Those exogenous fatty acids must be delivered with a diet [46]. PUFA synthesis occurs in liver which supplies them to the brain, owing to the brain’s little capacity for PUFA synthesis. LA is converted to γ-linolenic acid (GLA, 18:3, ω -6), dihomo-GLA (DGLA, 20:3, ω -6), and AA by liver’s desaturases and elongases [46]. Similarly, ALA is converted to eicosapentaenoic acid (EPA, 20:5, ω -3) and DHA via the same enzymatic route. Therefore, both liver enzymatic activity connected with the diet are im determinants of DHA and AA content in brain [46]. The important role played by the oleic acid is connected to SCD activity that catalyzes formation of double bonds into short-chain of FA in the C9 position [66]. It is known that this step changes the physicochemical properties of FAs and has profound effects on lipid function [41]. Recently we have shown that epithelial cells of the normal breast duct are saturated by oleic acid and/or its derivatives that are glyceryl trioleate and carotenoids. In contrast, the cancerous duct displays significantly smaller amount of monounsaturated fatty acids and triglycerides than the epithelial cells of the normal one [71]. The same profound effect of altered fatty acids composition is observed in brain tumor [72] as it has been also demonstrated by the results presented in this paper. It has also been observed that SCD activity is upregulated in certain types of tumors. It has been speculated that inhibition of SCD function may cause tumor cells death, by inducing the uncontrollable accumulation of unsaturated fatty acids [73] The reduced Raman intensity of saturated bonds at 1437-1444 cm^-1^ (proportional to the concentration) in brain tumors that we observe in our results may be related to enhanced production of polyunsaturated acids (like AA and its eicosanoid metabolites (PGs, leukotriens)) and /or down-regulated production of OA. The Figure 8 seems to suggests that the down-regulated production of OA occurs in brain tumors. The reduced Raman intensity of saturated bonds at 1437-1444 cm^-1^ (proportional to the concentration) in CNS tumor may be also partially related to increasing fatty acid degradation in cancer cells by increasing the rate of mitochondrial β-oxidation.

**Figure 9:**
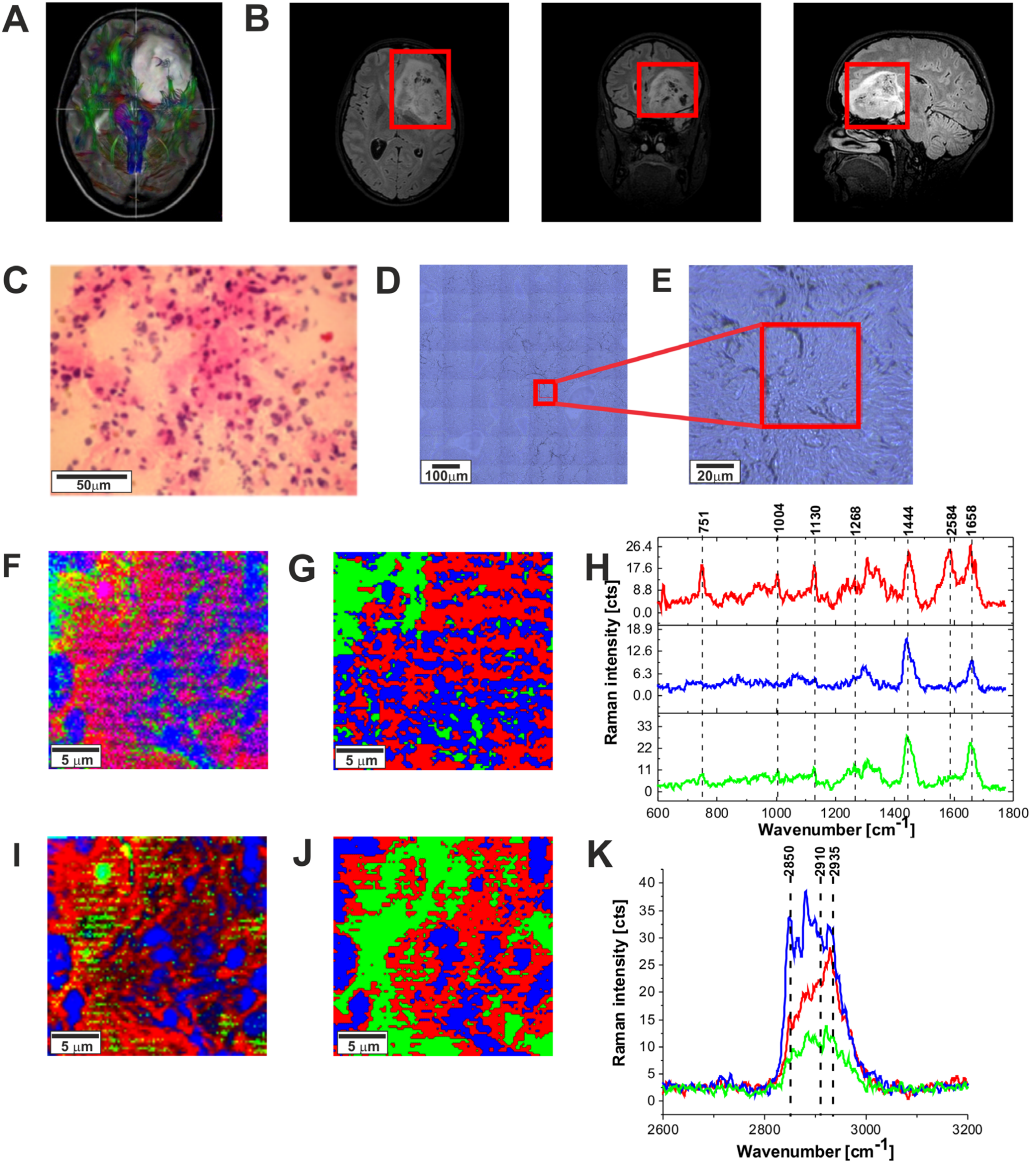
The tractography image **(A),** MRI image **(B),** H&E-stained histological image **(C), s**titching microscopy image (550 μm x 550 μm) **(D),** microscopy image (120μm x 120 μm) **(E),** Raman image (50 μm x 50 μm) by basis analysis **(F)** and cluster analysis **(G)** and the characteristic vibrational Raman spectra in the fingerprint frequency region **(H)** Raman image (50 μm x 50 μm) by basis analysis **(I)** and cluster analysis **(J)** and the characteristic vibrational Raman spectra in the high frequency region **(K)** of the tumor (Astrocytoma fibrillare, grade WHO II) (P9), The line colors of the spectra correspond to the colors of the Raman maps. Raman integration time for images 1s for low frequencies and 0.5 s for high frequencies, resolution step: 1 μm, laser excitation power: 10m W.

**Figure 10:**
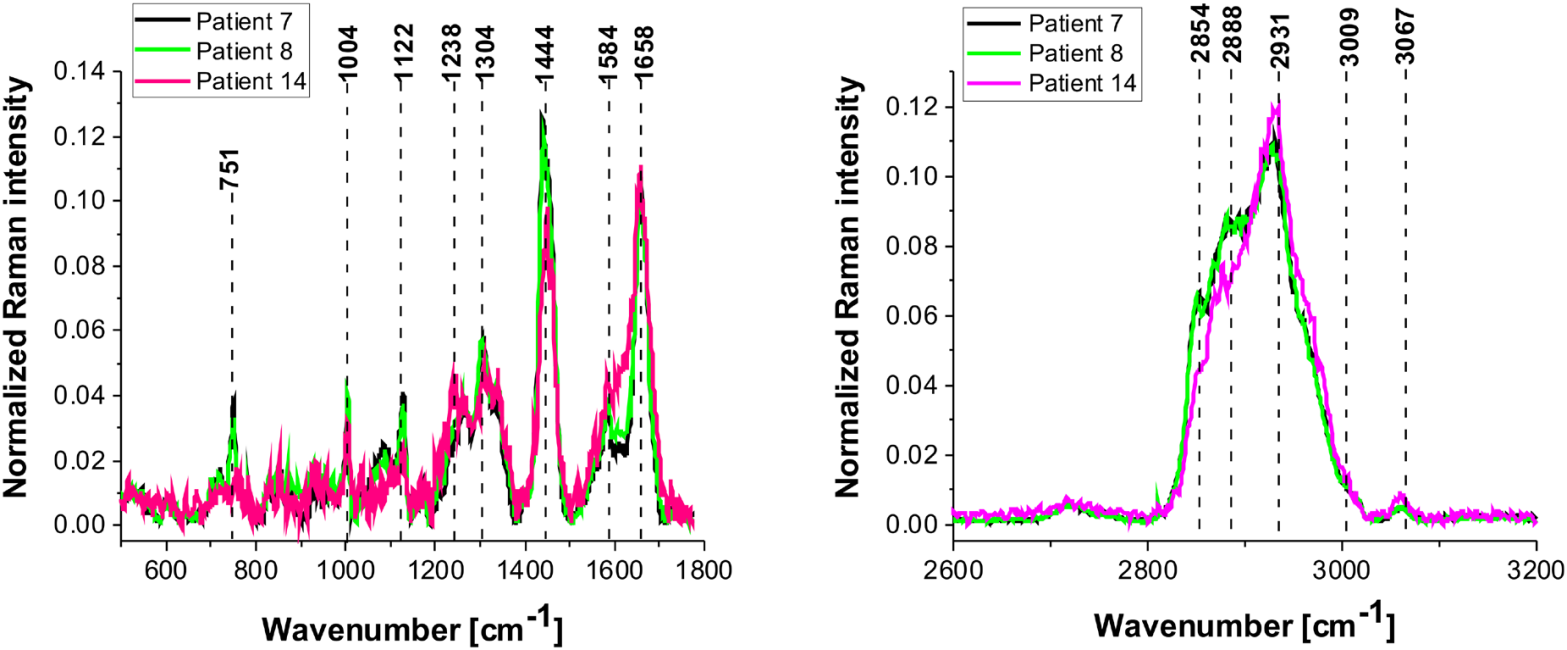
The average vibrational Raman spectra in the low and high frequency region for different areas of the low-grade brain tumor (astrocytoma, grade WHO I and II) (P7, P8, P14), Raman integration time for images 0.5 s for high frequency and 1s for low frequency region, resolution step: 1 μm, laser excitation power: 10m W

It is interesting to compare the results presented so far with the results for the low grade CNS tumors. Figure 9 shows the average Raman spectra in the fingerprint region for the low-grade astrocytoma compared with the spectra for normal CNS. Overall, the Raman spectra for low-grade CNS tumors appear to show many similarities and overlapping with the normal tissue Raman spectra. However, detailed inspection shows notable differences in the region 852-964 cm^-1^ (tyrosine, proline, glycogen, carbohydrates, collagen, glucose, lactic acid.), 1030 cm^-1^ (phenylalanine), 1338cm^-1^ (Tryptophan), C_α_-H def), 1441 cm^-1^ (fatty acids, triglycerides, CH_2_ or CH_3_ deformations). The enhanced level of tryptophan has been reported earlier [74, 75] in studies on primary gliomas; primary CNS tumors showing high levels of tryptophan uptake using the alpha-[(11)C] Methyl-L-tryptophan (AMT) as a PET tracer combined with magnetic resonance imaging (MRI) [74]. Similarly, Kamson *et al* [75]. suggest that the AMT-PET tracer coupled with MR imaging has the potential to differentiate between high-grade gliomas and CNS metastases, something which is difficult using conventional MRI [75]. When we compare the differences between the high grade tumor and the normal CNS tissue in Figure 6C and 6D with the differences for the low-grade astrocytoma in Figure 10 we do not observe the spectacular conformational modifications indicating that α-helix structure is not modified to β-sheet as a result of low grade brain tumor as it was observed for the high grade tumor presented in Figure 6.

## MATERIALS AND METHODS

### Study participants

**Chart 1:**
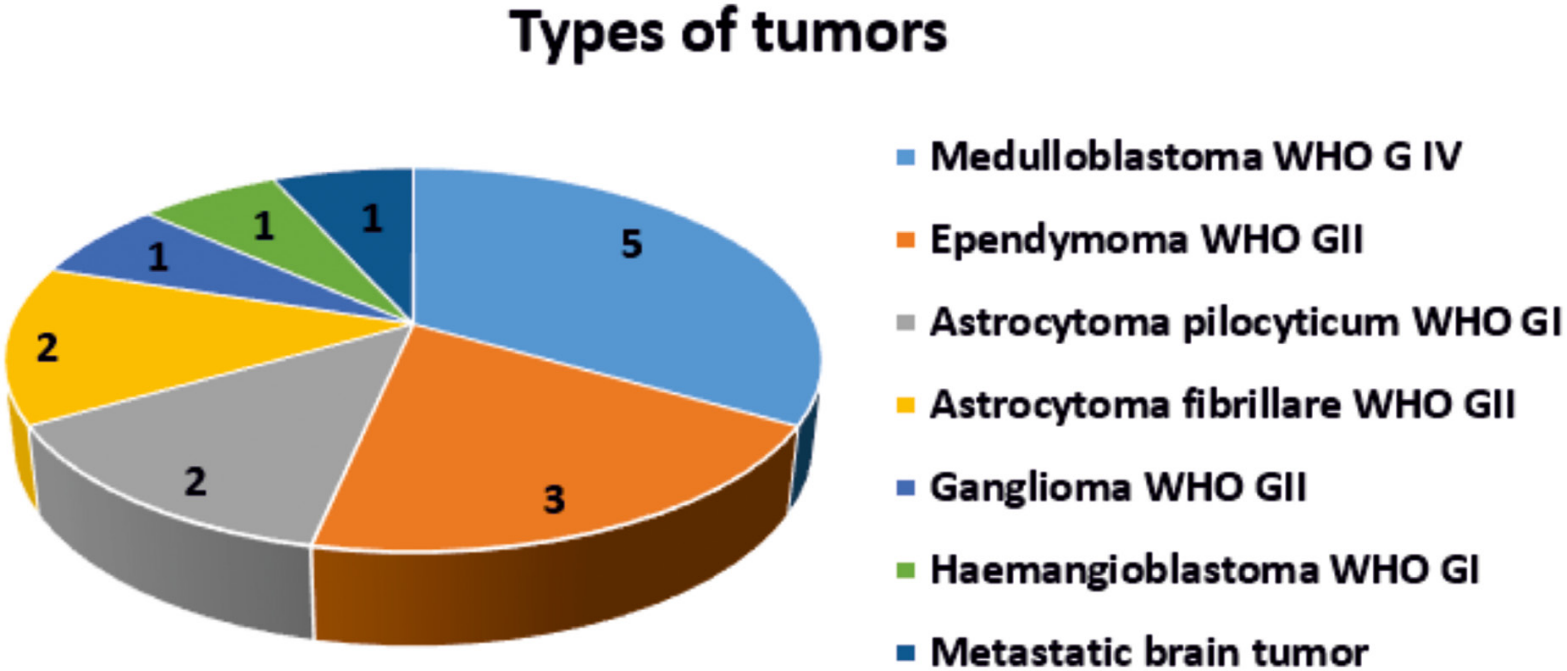
Types of brain tumors

All procedures have been conducted under a protocol approved by the Bioethical Committee at the Polish Mother’s Memorial Hospital Research Institute in Lodz (53/216). All experiments were performed in compliance with relevant laws and guidelines of the Bioethical Committee at the Polish Mother’s Memorial Hospital Research Institute in Lodz (53/216) and of the Ministry of Health of the Republic of Poland. Written informed consent was obtained from patients. The tissue samples consisted of medulloblastoma (grade IV) (n= 5), low-grade astrocytoma (grades I-II) (n =4), ependynoma (n=3) and metastatic brain tumors (n= 1). The tissue from the negative margins were used as normal controls (chart 1).

### Tissue preparation for Raman spectroscopy

Microtomed 16-μm-thick tissue sections were obtained from frozen blocks of the material removed at the time of surgical operation at the Polish Mother’s Memorial Hospital (Lodz, Poland) and placed on CaF_2_ substrates (CRYSTAL GmbH, Germany) for Raman spectroscopy and Raman imaging measurements. Parallel 6-μm tissue sections were obtained and stained with H&E followed by histology examination for all the specimens by certified neuropathologist from the Polish Mother’s Memorial Hospital Research Institute in Lodz. The tissue sections were interrogated by the Raman spectroscopy and Raman imaging. The single spectra were performed in a randomized fashion to avoid bias. We used fresh tissue instead of commercially available formalin-fixed paraffin-embedded (FFPE) tissue blocks because we have shown [22] that the lipid phenotype of cancer is disturbed in FFPE samples due to tissue processing (de-waxing, washing and clearing with solvents (e.g. xylene)) has essential effect on loss of cellular lipid constituents. Detailed methodology is available elsewhere [40].

### Raman spectroscopy and Raman imaging

We interrogated CNS both with the MRI imaging and Raman imaging. MRI images were used for visualization and location of tumor region for each patient. Detailed analysis of the tumor mass was performed by Raman imaging having much higher spatial resolution than MRI. Raman imaging measurement were performed on the preoperative MRI images. To explore the morphological and biochemical heterogeneity within brain tumors Raman images were generated by a confocal Raman microscope – WITec alpha 300 RSA (Ulm, Germany) consisting of an Olympus microscope, coupled with a 300 mm Czerny–Turner monochromator (Princeton Instruments Acton SP23000-300 mm Imaging Triple Grating Monochromator/Spectrograph) and a thermoelectrically cooled CCD Camera ANDOR Newton DU970N-UVB-353 (EMCCD chip with 1600 × 200 pixel format, 16 μm dimension each) operating in the standard mode at −64 °C with full vertical binning. The excitation laser beam was a second harmonic of the Nd:YAG laser (532 nm) which was focused on the sample with a 40× magnification objective (NIKON CFI Plan Fluor C ELWD 40×: NA 0.60, WD 3.6–2.8 mm; DIC-M, C.C.0-2) to the laser spot of 1 μm determined by the laser wavelength and microscope objective being used. The average laser excitation power was 10 mW, with a collection (integration) time of 0.5 s and a spectral step of 2 cm^−1^ in the fingerprint range of 200–1800 cm^−1^ and high frequency region of 1800-3600 cm^-1^. A piezoelectric table was used to record Raman images. The spectra were collected at one acquisition per pixel and a 1200 lines mm^−1^ diffraction grating with the spectral bandpass varying from about 5.5 cm^−1^ per pixel at about 200 cm−1 to about 3.3 cm^−1^ per pixel at 3600 cm^−1^. Raman images (50 × 50 μm, 100 × 100 points per line) from the fingerprint spectral regions of the human brain tissue from the tumor mass and from the safety margin were constructed.

Detailed methodology on data pre-processing and multivariate data analysis is used in the paper is available elsewhere [29, 76, 77].

The collected Raman data were preprocessed with the WITec Control/Project Plus 1.6/Project 2.10 package. The raw Raman data were corrected for cosmic rays. We used spatial and frequency filtering to remove sharp spikes attributed to cosmic rays. The cosmic rays corrected data were smoothed by a Savitzky and Golay procedure. Rayleigh scattering and the Raman spectrum of the CaF_2_ support was removed by the reduction of the spectral range to 400–1800 cm^−1^ before a multivariate analysis. The backgrounds were also subtracted before further analysis. The spectra were vector normalized (divided by norm). The visualization of chemical similarities and differences was also demonstrated in the human breast tissue images. The 2D array of tens of thousands of individual Raman spectra recorded from the human tissue from the safety margin and the tumor mass have been used to construct Raman images using the mapping function of the WITec Control/Project Plus 1.6/Project 2.10 package. K-means clustering analysis (KMCA) and basis analysis with Manhattan metrics have been employed to analyze Raman spectroscopic maps of human brain tissue. KMCA is a method of analysis which clusters n spectra of a dataset into k clusters such that each spectrum belongs to the cluster with the nearest (closest) mean. We used also the average basis spectra from characteristic regions of proteins, lipids, and nucleic acids using the specific spectral filters corresponding to Raman vibrations of these components. These values of the weighting factors are converted to a monochrome intensity map. The color code of the Raman maps was based on the integrated Raman intensities in specific regions (sum option in the filter manager in the WITec Project Plus 1.60). Using a lookup table, bright yellow colors indicate the highest intensities, whereas brown colors indicate the lowest intensities in a chosen region. Up to 9 monochrome maps for different weighting factors can be combined to yield a pseudo-color map with mixed colors indicating the intensity values of each of the weighting factors. The color maps can then be constructed to visualize the distribution of the weight factor magnitudes that provide information about the biochemistry of the molecules in terms of their vibrational frequencies and intensities. There are others reconstruction methods that may provide some advantages over PCA, but here we didn’t applied them [78].

## CONCLUSIONS

We have found that metabolism of proteins, nucleic acids, and lipids is markedly deregulated in malignant medulloblastomas. Our results indicates marked metabolic differences between high grade medulloblastoma and normal brain tissue. The Raman spectra for high-grade brain tumors show many significant differences compared to the normal brain spectra. Detailed inspection shows notable differences in vibrations of proteins, lipids, and nucleic acids:

1) the high-grade tumors of central nervous system (medulloblastoma) exhibit enhanced level of β-sheet conformation and down-regulated level of α-helix conformation when comparing against normal tissue.

2) almost all tumors studied in the paper have increased Raman signals of nucleic acids. This increase can be interpreted as increased DNA/RNA turnover in brain tumors.

3) The ratio of Raman intensities I2930/I2845 at 2930 and 2845 cm-1 is a good source of information on the ratio of lipid and protein contents. We have found that the ratio reflects the different lipid and protein contents of tumorous brain tissue compared to the non-tumor tissue. Almost all brain tumors have the Raman intensity ratios significantly higher than that found in non-tumor brain tissue, which is 1.456 ± 0.016, and indicates that the relative amount of lipids compared to proteins is significantly higher in the normal brain tissue. We found that levels of the saturated fatty acids were significantly reduced in the high grade medulloblastoma samples compared with non-tumor brain samples and low grade astrocytoma. Differences were also observed in the n-6/n-3 PUFA content for medulloblastoma and non-tumor brain samples. The content of the oleic acid (OA) was significantly smaller in almost all brain high grade brain tumors than that observed in the control samples. It indicates that the fatty acid composition of human brain tumors is different from that found in tumor-free brain tissue.

The iodine number NI for the normal brain tissue is 60. For comparison OA has 87, DHA 464, ALA 274. The high grade tumors have the iodine numbers between that for palmitic acid, stearic acid, arachidic acid (N_I_=0) and oleic acid (N_I_=87). Most low grade tumors have N_I_ similar to that of OA. The iodine number for AA (N_I_=334) is much higher than those observed for all studied samples.

An decrease in OA has been found in malignant CNS tumor (medulloblastoma) suggesting that tumor development is connected with enhanced OA loss/metabolism to the extent to the level to the brain OA level cannot be compensate by liver production or diet intake.

The low-grade astrocytoma does not exhibit spectacular conformational modifications indicating that α-helix structure is not modified to β-sheet as a result of low grade brain tumor as it was observed for the high grade brain tumors.
